# Different Stationary Phase Selectivities and Morphologies for Intact Protein Separations

**DOI:** 10.1007/s10337-016-3168-z

**Published:** 2016-09-23

**Authors:** A. Astefanei, I. Dapic, M. Camenzuli

**Affiliations:** Centre for Analytical Science in Amsterdam (CASA), Van’t Hoff Institute for Molecular Sciences, University of Amsterdam, Science Park 904, 1098 XH Amsterdam, The Netherlands

**Keywords:** LC–MS, Liquid chromatography, Top-down proteomics, Intact proteins

## Abstract

The central dogma of biology proposed that one gene encodes for one protein. We now know that this does not reflect reality. The human body has approximately 20,000 protein-encoding genes; each of these genes can encode more than one protein. Proteins expressed from a single gene can vary in terms of their post-translational modifications, which often regulate their function within the body. Understanding the proteins within our bodies is a key step in understanding the cause, and perhaps the solution, to disease. This is one of the application areas of proteomics, which is defined as the study of all proteins expressed within an organism at a given point in time. The human proteome is incredibly complex. The complexity of biological samples requires a combination of technologies to achieve high resolution and high sensitivity analysis. Despite the significant advances in mass spectrometry, separation techniques are still essential in this field. Liquid chromatography is an indispensable tool by which low-abundant proteins in complex samples can be enriched and separated. However, advances in chromatography are not as readily adapted in proteomics compared to advances in mass spectrometry. Biologists in this field still favour reversed-phase chromatography with fully porous particles. The purpose of this review is to highlight alternative selectivities and stationary phase morphologies that show potential for application in top-down proteomics; the study of intact proteins.

## Introduction

Proteomics is often applied to clinical studies in the search of biomarkers [1]. These biomarkers are mostly proteins that are found in the tissue or plasma of patients suffering from a particular disease yet may be expressed in different amounts in healthy patients. While this sounds simple, the reality is that there are approximately 20,000 protein-encoding genes in the human body [2]. Many of these genes code for more than one protein isoform (proteoforms). These proteoforms arise from various post-translational modifications (PTMs) including phosphorylation, methylation and ubiquitination to name but a few, which can change the function of the protein in addition to modifying its structure. Since there are a number of amino acids that can act as sites for PTMs it follows that proteoforms can have varying degrees of PTMs in addition to multiple types of PTM. Consequently from any given protein-encoding gene, a large number of proteins can be produced. Proteins are expressed in varying abundance with some proteins such as albumin in blood, being much more abundant than other proteins present in biological material. It follows that these aspects significantly complicate the study of any proteome. Such samples require analytical techniques capable of providing high resolving power and sensitivity.

Mass spectrometry (MS) is an obvious choice for proteomics research given its separation power and the ability to characterize protein structure through the interpretation of the fragmentation patterns in mass spectra. However, the analysis of intact proteins using MS faces a number of technical challenges. The large dynamic range in protein abundances within a sample can result in the suppression of the ionization of low abundant proteins, reducing their ability to be detected. Once ionized, intact proteins feature multiple charge states all corresponding to the same protein species, with multiple isotopes for each charged state. Different types of mass spectrometers present different levels of resolving power which may or may not be enough for the isotopic distributions of each protein multiple charge states to be resolved. Developments in Fourier Transform Ion Cyclotron Resonance (FTICR) MS has been an important step towards improving our ability to analysis proteins. In an MS imaging application using FTICR and secondary ion MS, resolving power in the order of 3,000,000 has been reported [3]. This compares to a resolving power of 2000–10,000 using time-of-flight (TOF) in a similar setup.

Even with the high resolving power of FTICR MS, hyphenation of MS with other separation techniques is necessary to reduce the sample complexity. Liquid chromatography (LC) is widely used for this purpose due to its high separation power and the ability to hyphenate it with MS, typically via electrospray ionization (ESI). However, using LC for protein separations faces its own technical challenges. Proteoforms often have similar physio-chemical properties making their separation extremely difficult. In addition the diffusion coefficient of proteins is relatively small compared to small molecules, increasing the time taken for mass transfer resulting in chromatographic band broadening. Furthermore, proteins can be difficult to dissolve in solvents commonly used as mobile phase in LC. These challenges can be avoiding by digesting the proteins into peptides using enzymes such as trypsin. This approach is commonly referred to as “bottom-up”. The bottom up approach is not without its own pitfalls, namely the possible loss of protein information [4, 5]. Digestion of proteins into peptides can result in peptides whose sequence is present in a number of proteins, making protein identification prone to error. Missed cleavages due to inefficient digestion can hamper the ability for bioinformatics software to match peptide sequences derived from mass spectra to the sequences within software databases. The potential loss of information can include valuable information such as the position of PTMs that are of interest in the study of biological pathways. The analysis of intact proteins, referred to as “top-down” can avoid the pitfalls of the bottom-up approach if sufficient fragmentation of protein ions can occur in the gas phase during tandem MS (MS/MS) [4]. It follows that the top-down approach is attractive for applications where complete information on the protein structure is of primary importance. This requires the technical challenges of separating intact proteins with LC to be addressed.

To address the challenges faced by top-down proteomics, a range of different chromatographic selectivities and stationary phase morphologies can be employed. However, the proteomics community still remains largely faithful to reversed phase chromatography (RPLC) using C18, C8 or C4 stationary phases and fully porous particle packed columns. The latest developments in column technology, such as core–shell particles and monolithic columns appear to remain obscure to most scientists within the field of proteomics. Relatively new selectivities such as hydrophilic interaction liquid chromatography (HILIC) are becoming more recognised, namely for the analysis of glycoproteins [6, 7], yet are not widely applied. This bottleneck in knowledge transfer between the analytical and biological communities, in part, may be due to the rapid advancement of MS technology in addition to the abundance of reviews and research publications focusing on the use of MS; we cite just a few recent reviews here for those readers seeking the MS perspective on protein analysis [5, 8–11]. The purpose of our review is to address this imbalance by presenting recent advancements in LC column technology for protein separations. We will discuss the selectivities and column morphologies most recently used in addition to emerging chromatographic technologies that have shown potential value for the separation of intact proteins.

## Implementation of Different Selectivities in Intact Protein Analysis

Proteomic studies include different approaches such as protein profiling, monitoring PTMs and protein–protein interactions. Each of these application areas requires a certain type of chromatographic separation. RPLC has a long tradition in intact protein analysis and its compatibility with electrospray ionization MS has made it an important technique in top-down proteomics. However, other separation modes that separate based on protein structure, mass, charge or the presence of specific chemical functional groups are also employed. These techniques include hydrophilic interaction liquid chromatography (HILIC), affinity chromatography, hydrophobic interaction chromatography (HIC) and size exclusion chromatography (SEC).

### Reversed-Phase Liquid Chromatography

RPLC is one of the most widely used methodologies in protein analysis [12]. Structural features of proteins such as their conformation, size and molecular weight make RPLC of intact proteins more demanding compared to small molecule separations in terms of carry-over, peak broadening, multiple peak formation and strong adsorption on the stationary phase [13–15]. To improve chromatographic performance in RPLC many parameters such as particle size, mobile phase, column temperature and column length can be optimized. Due to their hydrophobic properties, proteins show strong retention on long chain (C8, C18) stationary phases that leads to their low recovery, peak tailing and a decrease in intensity. Therefore, less hydrophobic stationary phases with shorter alkyl chains (e.g. C2, C4) that show faster desorption of proteins from the stationary phase are preferable. The power of RPLC for analysis of proteins was demonstrated by Rehder et al. for the separation of the light chain and two variants of heavy chains (N-terminal glutamine and N-terminal pyroglutamate) of reduced monoclonal antibodies [16]. For the separation different stationary phases including C3, C8, C18, and CN Agilent Zorbax Stable Bond SB300 columns with 3.5 μm particle size and the Varian Pursuit DiPhenyl with 3 μm particle size were tested with an increasing percentage of *n*-propanol in acetonitrile and 0.1 % trifluoroacetic acid (TFA) was used as the mobile phase. The Varian DiPhenyl column showed highest plate number while Zorbax CN column showed highest selectivity and resolution.

**Fig. 1:**
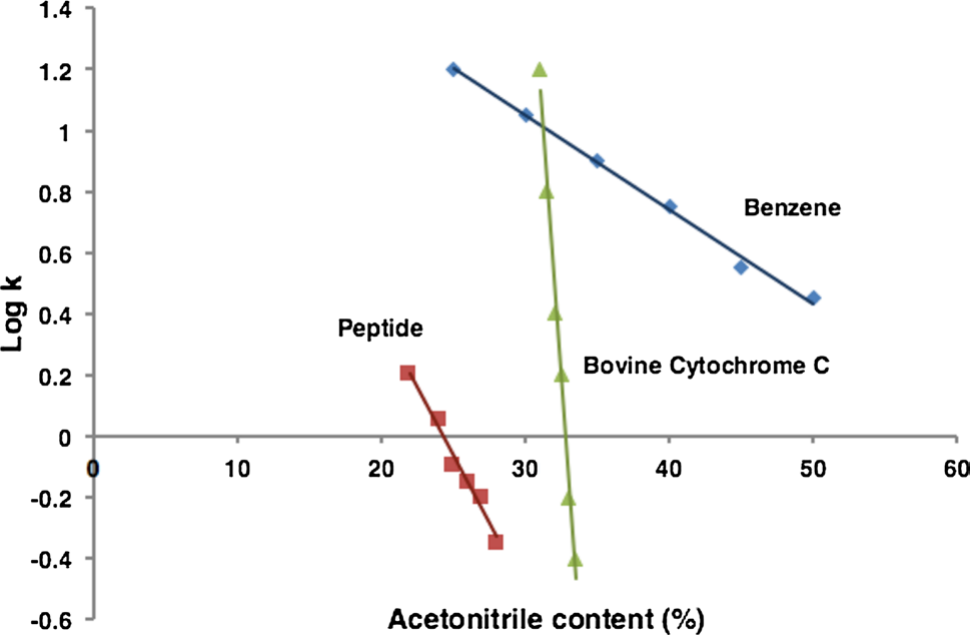
Relationship between the retention factor and the proportion of organic modifier (acetonitrile) in the mobile phase, shown for a small molecule (benzene, represented by *diamonds*), a peptide (*squares*) and a protein (bovine cytochrome C, *triangles*) Adapted from [17]

Faster desorption of proteins from the stationary phase can also be achieved using gradient elution. It is well known that in RPLC the retention of the analytes decreases with the increase in the proportion of the organic solvent in the mobile phase. For proteins, the change in retention with increasing organic modifier content is much more significant than for small molecules (Fig. 1) [17, 18]. Therefore, small increases in organic modifier lead to large reductions in retention amounting to what may appear as an ‘on–off’ retention mechanism. Another important advantage to using gradient elution is peak compression. In brief, peak compression occurs because the rear part of the solute band (or peak) is eluting faster than the front part of the solute band. This occurs because of the increasing elution strength of the mobile phase as a function of gradient time [19]. The resulting peak compression reduces the peak width counteracting the effects of band broadening. Mobile phase additives, such as TFA, also effect retention and peak shape. Recently the effect of a wide range of common mobile phase additives was examined for 11 intact proteins [20]. While the use of TFA provided symmetrical peak shapes (due to ion-pairing), it was linked to a loss in MS sensitivity attributed to ionization suppression. The use of 10 mM formate buffer (pH 3) was a suitable MS-compatible alternative to TFA providing a boost in sensitivity by a factor of 5 compared to TFA, without compromising peak shape.

Together with the physical properties of the stationary phase other aspects such as pressure, temperature and the composition of the mobile phase, play important roles in governing the success of intact protein separations. The introduction of smaller particles and ultra-high performance liquid chromatography (UHPLC or UPLC) means that protein separations can now occur at pressure over 1000 bar. In the study of Eschelbach et al. the effect of elevated pressure on protein recovery and protein carry-over for 4 model proteins ribonuclease A, ovalbumin, myoglobin and bovine serum albumin (BSA) was examined [14]. Results showed that for separations of 4 proteins in 5 μm packed column and at pressure of 160 bar carry-over was present and examining mass spectra indicated that it was necessary to run 6 blanks to clean the column. Inspection of mass spectra from the separation of the same proteins on the 1.4 μm packed column and at pressure of 1580 bar indicated that there was no carry-over present. While this result is encouraging, it is important to note that pressure affects the way that proteins are retained on the stationary phase which means that attention must be paid when transferring separation methods from lower pressure to higher pressure. Retention changes under pressure arise from a change in the molar volume of proteins. The theory behind this mechanism is quite complex as the effect of pressure on retention is intertwined with temperature [21] and the change of protein conformation upon adsorption onto the stationary phase and the kinetics of this adsorption [22]. In brief increasing pressure reduces the solvation layer of the alkyl-bonded phase of C18 stationary phase. In conjunction to reducing the solvation of the ligands, solvent molecules form a solvation layer around the protein. It is known that proteins change conformation, exposing their hydrophobic regions, upon adsorption onto the stationary phase in reversed phase conditions [23]. Exposure of the protein’s hydrophobic region in conjunction with a reduction in the solvation of the protein and the possible change in the conformation of the stationary phase ligands increases the retention in reversed-phase conditions. This increase in retention in conjunction with pressure has been shown to increase retention as high as 300 % for insulin variants [22]. Such gains in retention were not as large for other proteins [24] indicating that the effect of pressure is protein-specific.

Effects of pressure on retention have been shown to be interrelated to the effects of temperature on retention [21, 24]. There are two sources of temperature in chromatography: frictional heating generated as mobile phases passes through the stationary phase at high linear velocities, under high pressure conditions such as those found in UHPLC and heating of the column and mobile phase using column heating devices. As the mobile phase travels through the stationary phase under these conditions, friction occurs and generates radial and axial temperature gradients [25]. These temperature gradients generate viscosity gradients as a result of the relationship between viscosity and temperature. The consequence is the formation of radial linear velocity gradients that distort the solute band as it moves through the column, reducing the efficiency of the separation for small molecules. While the relationship between efficiency and frictional heating has not been defined for proteins, its effect of retention has been studied. At constant inlet pressure, thereby reducing the effect of pressure, frictional heating of 1.5–1.7 W heat power reduced the retention of insulin by 20–45 % [26]. For larger proteins, namely myoglobin and lysozyme, the effect was more significant: a reduction in retention by up to 75 % [24]. While the effect of frictional heating arises from the generation of heat within the column, applying temperature externally by heating up the column and mobile phase also affects retention for small molecules and proteins. For small molecules, increasing temperature is known to increase the rate of diffusion of the solute molecules, speeding up their mass transfer and thereby gains in efficiency can be seen with a change in the value of the optimum flow rate. The application of temperature and pressure is expected to cause denaturation of the protein, that is, the unfolding of the protein structure. Given that different proteins have a different ratio of secondary structural elements, such as α-helices and β-sheets, the relationship between denaturation and protein conformational changes may be protein-specific. The protein-specific nature of the relationship between temperature and retention was shown for filgrastim, interferon alpha-2A, lysozyme and myoglobin [24]. At a constant pressure of 200 bar, retention decrease significantly with increasing temperature for lysozyme and myoglobin from retention factors of around 6 at 20 °C to less than 2 at 70 °C. Filgrastim and interferon alpha-2A showed a different relationship: retention increased with increasing temperature until a specific temperature range around 50 °C after which retention started to decrease. In general, these relationships were seen at other constant pressures up to 1000 bar with some amplification of the effect owing the relationship between pressure and retention, where retention increased with increasing pressure. Denaturation exposing the hydrophobic core of proteins does agree with the increase in retention initially seen for some proteins in reversed phase conditions. However, the observed decreasing trend in retention with increasing temperature past a certain value seems to contradict this and suggests perhaps another factor is influencing retention other than exposure of the hydrophobic core. A decrease in the molar volume of the protein at temperatures higher than 50 °C has been put forward as an explanation of the decreasing retention at elevated temperatures.

### Hydrophilic Interaction Liquid Chromatography (HILIC)

HILIC presents powerful alternative for separation of proteins that show strong retention in RPLC. Advantages such as selectivity towards highly polar compounds, reduced backpressure due to the higher amount of organic solvent used compared to RPLC and increased LC–MS sensitivity have become important aspects in use of HILIC in analysis of proteins. The retention mechanism of HILIC, characteristics of stationary and mobile phases and also applications have been the topic of several recent reviews [27–32]. HILIC alone or in combination with other techniques has been used for the desalting and fractionation of proteins prior to MS, analysis of membrane proteins, or study of protein PTMs [6, 33–35]. The advantage of HILIC is that by utilizing a polar stationary phase it promotes the retention of polar compounds, which are then eluted using isocratic or gradient elution by increasing the hydrophilicity of mobile phase by increasing the proportion of polar aqueous solvent. Therefore in HILIC, hydrophilic compounds elute later than hydrophobic compounds and the elution order is typically the opposite to their elution in RPLC. However, high amounts of organic solvents used in HILIC chromatography could cause protein denaturation, which limits its use in native protein analysis. Moreover, ion-pairing agents such as TFA are often added in mobile phase to improve peak shape and resolution and change the retention of the proteins. Addition of TFA can promote ion-pairing mechanism and retention of the proteins may be more driven by their hydrophilic residues and modifications. However, when mobile phase consisting of TFA is coupled to MS, analyte signal intensity might be reduced [20]. In the study of Periat et al., comparison of mobile phases containing 0.1 % TFA, 50 mM ammonium formate and 0.5 % acetic acid for the analysis of RNase B showed that TFA significantly reduced retention time in addition to enhancing peak shape and resolution compared to the other two solvents [34].

The retention mechanism in HILIC is considered to be a combination of partition and adsorption of compounds between the mostly organic mobile phase and the partially immobilized water layer on stationary phase. Interactions between analytes and the stationary phase are complex and can arise from the combined effect of electrostatic, hydrophobic and ion-exchange interactions while hydrogen bonding also might be involved. The dominant interaction depends on the type of stationary phase together with the pH and composition of the mobile phase. In HILIC mobile phases generally consist of 5–50 % water with respect to the proportion of water miscible organic solvents where the most common solvent used is acetonitrile [29]. Alcohols can also be used; however, a higher proportion of these solvents in the mobile phase is needed to provide similar retention of the analyte as when an aprotic solvent is used. Other suitable organic solvents can be selected based on the eluotropic series showing the relative elution strength of organic solvents used in HILIC, such as acetone < isopropanol ~ propanol < acetonitrile < ethanol < dioxane < DMF ~ methanol < water [28]. HILIC separations can be performed in isocratic or gradient elution mode. Isocratic elution usually consists of a high percentage of organic solvent in mobile phase while in gradient elution the starting composition of mobile phase gradient consists of high percentage of organic modifier and the elution is promoted by increasing percentage of water.

Diverse stationary phases are used in HILIC separations depending on application. Stationary phases often include highly polar functional groups similiar to those in normal phase LC such as hydroxy, amino, amido and cyano functionalities. [28]. New stationary phases for HILIC are continuing to be developed and applied for protein separations. Carrol et al. reported the use of a polyhydroxyethyl-aspartamide HILIC column for the separation of mitochondrial membrane proteins [36]. An evaluation of different HILIC columns was carried out by Tetaz et al. where they compared four HILIC stationary phases: Polyhydroxyethyl A (silica coated with polyhydroxyethylaspartamide), ZIC-HILIC PEEK (zwitterionic ligand covalently attached to porous silica), ProntoSIL 300-5-Si (bare silica) and TSKgel Amide 80 (polymer coated silica) for the separation of human apoA-I, recombinant human apoM and equine cytochrome C [6]. It was shown that human apoM and human apoA-I eluted later using ZIC-HILIC PEEK column compared to polyhydroxyethyl A under the same mobile phase conditions. Electrostatic interaction of analyte with the zwitterionic ligand on the ZIC-HILIC column might be a reason for later elution of the aforementioned proteins and for non-elution of cytochrome C within 30 min.

HILIC has proven useful for addressing one of the most challenging PTMs; glycosylation. Due to complexity emerging from microheterogeneity of sugar moieties, RPLC of intact glycoproteins often does not show enough resolution for distinguishing between different glycoforms. Therefore, the potential of HILIC for the analysis of glycans while they are still attached to protein backbone as complementary approach to analysis of released glycans has been examined [37]. Pedrali et al. showed potential for separation of ribonuclease A and five intact isoforms of its naturally glycosylated variant ribonuclease B on a HILIC amide column [37]. Moreover, HILIC chromatography has showed large potential in analysis of histone forms from human cells [38] and H4 forms from the HeLa cell lines [39]. Histone separation can be achieved by the separation of their subtypes based on the number of acetyl groups and then by the level of methylation, greatly reducing sample complexity. In the study of Pesavento et al. the combination of RPLC and HILIC was used to separate different forms of histones [35]. Histone H4 was first purified from crude HeLa S3 histone using RPLC and multiply modified histone H4 forms were then separated using HILIC PolyCAT A column with each 1 min fraction analyzed separately using Fourier transform MS. Fractions eluted according to the degree of their acetylation or methylation with the most hydrophobic fractions (tetra-acetylated and triple-methylated fractions) eluting first compared to N-terminal acetylation only allowing 42 unique histone forms to be characterized and quantified.

### Affinity Chromatography

Another effective technique for protein separation, enrichment and purification is affinity chromatography which is based on reversible adsorption of targeted protein to the ligand that is immobilized on to the matrix [40]. The possibilities affinity techniques provide for rapid purification of proteins, high protein loading, compatibility with different buffers and additives have made them popular. Typical workflows include the use of conditions that favour maximum protein adsorption from sample loading, the washing step for removal of unbounded substances and desorption of the target protein from the column in elution step. Binding of the protein to ligand can be influenced by several parameters such as the amount of targeted protein and immobilized ligand, the flow rate used for binding and the nature of protein–ligand interaction. Higher flow rates might reduce protein binding if the interaction protein–ligand is weak or mass transfer-rate is slow [41].

One of the most widely used affinity techniques used for intact proteins is immobilized metal affinity chromatography (IMAC). This technique is based on the affinity of certain amino acid residues like cysteine, tryptophan and histidine exposed on protein surface for binding with metal ion coordination sites [40]. The metal ion is covalently attached to a chelating agent that is immobilized on the stationary phase surface and together they form an immobilized metal ion chelate complex. Retention of proteins can be affected by the nature of the metal ion, the structure and density of chelating compound, the presence of salt and additives in the buffer, organic solvent and protein size. Adsorption of proteins is based on interaction of electron donor groups located on protein surface and the immobilized metal ions that act as an electron pair acceptor. Interactions between metal ions and proteins are complex and have been shown to be combination of electrostatic (or ionic), hydrophobic and/or donor–acceptor coordination interactions [42]. The most commonly used are transition metal ions: Cu^2+^, Zn^2+^, Co^2+^, Ni^2+^ and Fe^2+^. Some electron-donor atoms such as N, S and O that are present in the chelating compounds attached to the support can coordinate metals. Remaining metal coordinating sites are mostly occupied by water or buffer molecules and can be exchanged with electron-donor groups from proteins. While many protein residues such as aspartic acid, histidine, glutamic acid, arginine, lysine, methionine, tyrosine and cysteine can participate in binding, the imidazole side chain of histidine residues as an electron-donor contribute more to binding than the N-terminus of proteins [40, 43].

The most commonly used solid supports in IMAC are based on soft-gel matrices such as agarose, cross-linked dextran or inorganic adsorbents like silica [40, 44]. Characteristics of the solid support and immobilization conditions should allow the maximum amount of protein to be adsorbed, show low non-specific adsorption, have uniform pore size and stability under wide range of experimental conditions. Low mechanical strength of gel matrices limits their use in high-pressure systems therefore other materials with better mechanical properties have been considered for use. Silica has the potential to be used as rigid support for fast and efficient separations and possesses higher mechanical strength compared to soft-gel matrices. However, its surface needs to be modified by coating with hydrophilic materials to minimize irreversible non-specific adsorption of proteins. Silica surface modifications can include adsorption of agarose, dextran or chitosan to reduce irreversible adsorption of the proteins. Another alternative to soft-gel matrices is the use of microporous membranes as supporting matrices since they show higher sample throughput and stability by allowing higher flow rates [45]. Immobilized Membrane Affinity Membrane (IMAM) phosphate Zr^4+^-IMAM was used for the evaluation of adsorption and selectivity of phosphorylated proteins (β-casein and ovalbumin) and non-phosphorylated proteins (bovine serum albumin and lysozyme). The adsorption isotherms showed that phosphate Zr^4+^-IMAM had higher binding capacity and selectivity for phosphorylated proteins compared to non-phosphorylated proteins. Adsorption of β-casein and ovalbumin increased in the range of protein concentration of 0.1–0.6 mg mL^−1^ while for BSA and lysozyme no significant increase was observed even at concentration of 1 mg mL^−1^ showing potential of use of phosphate Zr^4+^-IMAM for enrichment of phosphorylated proteins [46]. The development of new stationary phases for specific and selective binding of proteins and good protein recovery lead to IMAC being extensively used in antibody purification [47–51]. Evaluation of performance of iminodiacetic acid (IDA) and Tris(2-aminoethyl)amine (TREN) as a chelating agents in purifications of IgG with immobilized nickel affinity polyethylene vinyl alcohol (PEVA) hollow fiber membrane chromatography showed that Ni(II)-TREN had lower binding capacity for IgG compared to NI(II)-IDA; 9.8 and 9.4 mg for Ni(II)-IDA-PEVA and 1.4 and 1.5 mg Ni(II)-TREN for protein elution and regeneration, respectively [52]. Another study using tridentate (IDA), tetradentates (NTA, CM-Asp), and pentadentate (TED) chelate agents showed that using higher dentate agents increases selectivity in binding the proteins but show lower protein binding capacities compared to IDA [45].

Due to the ability of phosphate groups to chelate metal ions, IMAC has become important tool to enrich phosphorylated proteins prior to analysis with MS [53–57]. It has been shown that phosphorylated proteins prefer binding to Fe^3+^, Al^3+^ or Ga^3+^ [56, 58, 59]. While Fe^3+^ is typically used Machida et al. compared Ga^3+^, Fe^3+^, Zn^2+^ and Al^3+^ showed that Ga^3+^ proved to be the most efficient [59]. IMAC has proven to be an effective tool for comprehensive phosphoproteomic studies in plants. Enrichment of phosphoproteins using PHOS-Select iron affinity gel beads allowed detection of 132 phosphoproteins from *Arabidopsis* leaves. Depletion of d-ribulose biphosphate carboxylase/oxygenase (Rubisco) and other highly abundant proteins using polyethylene glycol (PEG) fractionation alone significantly increased number of identified phosphorylated proteins while in combination with IMAC more than double phosphorylated proteins were identified in depleted samples [56, 57]. A somewhat similar technique, metal oxide affinity chromatography (MOAC) has also been employed in phosphoproteomics [60]. In this technique, metal oxides such as aluminium hydroxide (Al(OH)_3_), titanium dioxide (TiO_2_) and zirconium dioxide (ZrO_2_) are typically used for the enrichment of phosphorylated proteins and more commonly, phosphorylated peptides [61–64]. In a recent study, the enrichment of phosphorylated proteins from a mixture of phosphorylated proteins (*β*-casein and ovalbumin) and non-phosphorylated proteins (BSA, myoglobin and cytochrome C) was preformed using ZrO_2_ nanofibers prepared by electrospinning [65]. Results demonstrated selective adsorption of acidic, neutral and basic phosphorylated proteins on the ZrO_2_ nanofibers when loading buffers of different pH were used.

While metal affinity techniques have proven the most popular for the protein analysis, other affinity techniques have also been used. An efficient tool for the collection of highly purified recombinant proteins is to use genetically engineered polyhistidine tags attached to the proteins of interest. The presence of multiple histidine residues improves binding of the protein to the support, usually containing Cu^2+^ or Ni^2+^. While the number of tags attached to the protein might vary depending on the study, the most popular is the His6 tag [66–68]. In one study, Magnusdottir et al. observed a tenfold increase in the yield of His6-GFP using IMAC for *Escherichia coli* lysate in which periplasm components were removed prior to lysis [69]. Aside from enrichment, using affinity techniques to remove specific proteins or protein groups has been proven to be an efficient way in overcoming the obstacle presented by the wide dynamic range of protein concentrations.

Depletion of highly abundant plasma proteins using different depletion techniques based on immunoaffinity protein removal enabled selective profiling of low-abundant proteins [70–72]. One of these methods, known as multiple affinity removal system (MARS) is based on presence of different high-affinity antibodies which are designed for rapid removal of high abundant proteins such as albumin, IgG, IgA, transferrin, haptoglobin and antitrypsin from human biological fluids [73, 74]. A step forward in technology was online immunodepletion in two dimensional systems where automatic depletion, desalting and fractionation was achieved [75]. The combination of MARS immunodepletion and multi-lectin affinity chromatography, M-LAC, was investigated for rapid screening of changes in protein levels in particular diseases [76]. Double separation of samples enabled the identification in changes in the level of the proteins angiotensinogen and apolipoproteinCl in patients compared to controls and might be used as a potential tool to reduce complexity of plasma samples. The limitation of immunoaffinity techniques is that they require antibodies with affinity to the protein of interest. It is not always feasible to acquire such antibodies, which may explain the relatively large number of applications of metal affinity techniques compared to immunoaffinity methods.

### Hydrophobic Interaction Chromatography

Using Hydrophobic Interaction Chromatography (HIC) native protein structure is more preserved in comparison to RPLC and is widely used in protein purification, often in combination with other chromatographic techniques [77–80]. In HIC protein separation is based on their hydrophobicity in non-denaturing mode with high resolution and selectivity that is orthogonal to RPLC [81–84]. Hydrophobic regions of the proteins interact with hydrophobic ligands from the stationary phase (butyl, octyl, phenyl) in conditions which promote hydrophobic interactions with the stationary phase, such as high concentrations of salt present in mobile phase [79]. In an aqueous medium, hydrophilic amino acids residues in the protein form hydrogen bonds with surrounding molecules and water molecules to form ordered structures around macromolecules. Addition of salts promotes solvation of salt ions and decreases number of water molecules interacting with hydrophilic regions of protein. Under these conditions protein molecules will have stronger intermolecular interactions and will self-associate or aggregate which is a thermodynamically favoured process [79, 85]. The impact of certain ions on hydrophobic interactions can be estimated using Hoffmeister series and optimal concentration of the salt for separation can vary due to individual differences in interaction between protein and stationary phase. High salt concentration promotes protein–ligand interaction and protein desorption is stimulated using gradient elution with decreasing salt concentration. The most commonly employed salts are sulfates, phosphates or citrates and by changing salt type and concentration in the mobile phase, protein retention can be manipulated (‘salt-promoted retention’) [86].

HIC columns are mostly based on silica or polymer particles; however, varying the support and ligand type has led to a wide range of stationary phases being developed [87]. Most commonly used are moderately hydrophobic ligands such as *n*-alkanes (butyl, octyl, phenyl) [82, 88]. However, newly developed materials have emerged which use cholesterol [89], dendronic ligands [90] and dual functional stationary phases [91]. Dual function HIC/strong cation exchange (SCX) silica-based stationary phase containing benzyl and sulfonic functional groups was used to separate seven proteins. The separation using this novel stationary phase showed to be comparable to the HIC column TSK-gel Ether 5PW and SCX PolyC columns when operating in HIC and SCX mode, respectively. Mass recoveries on SCX/HIC column for cytochrome C, RNase A, RNase B, lysozyme were more than 97 % in both modes while bioactivity for lysozyme was 96 and 98 % for HIC and SCX mode, respectively [91]. Ligand chain length, density and type of support or matrix are important aspects for consideration regarding the selectivity and the strength of interaction with the protein [79, 92]. However, protein retention also depends on the mobile phase composition (salt type and concentration, presence of additives), temperature and pH [93, 94]. Cusumano et al. evaluated the impact of different ligand chemistries (butyl, ether, alkyamide) for six different HIC columns: TSKgel Butyl-NPR, TSK gel Ether-5PW, Protein-Pak Hi Res HIC, MAbPac HIC-Butyl, MabPac HIC-10 and MAbPacHIC-20 and four different buffer systems (ammonium acetate, ammonium sulfate, sodium acetate and sodium chloride) [95]. Different stationary phases showed different selectivities towards the monoclonal antibody (mAb) mixture. When using sodium acetate buffer for mAbs analysis, HIC columns TSKgel Butyl-NPR and MAbPac HIC-Butyl showed the highest peak capacities while the TSK gel ether column showed the lowest efficiency. Using different buffer systems changed the retention and selectivity of mAb, showing that on HIC-10 column more than double the concentration of sodium acetate is needed to provide the same retention as with ammonium sulfate. The drawback of HIC is that the salts used in mobile phase are usually not compatible with MS and a desalting step is required. The presence of ammonium acetate as a MS compatible salt showed weak retention of proteins on polypropil A stationary phase [81]. Another possibility to increase protein retention was increasing the stationary phase hydrophobicity as demonstrated by Chen et al. who prepared new HIC materials (polypentyl A, polyhexyl A, polyheptyl A, polyoctyl A, polynonyl A and polyhydroxydecyl A) for protein elution using MS compatible concentrations of ammonium acetate (1 M or less) [82].

### Size Exclusion Chromatography

Size exclusion chromatography (SEC) is a technique used for the separation of proteins based on their molecular size (hydrodynamic volume) rather than on their chemical properties. It is widely used in analysis of protein biotherapeutics and monitoring protein aggregation [96–98] and its main advantages are mild elution conditions that have minimal impact on protein conformation and environment [99]. The separation of the biomolecules in SEC is based on the exclusion of proteins from the controlled particle pore sizes of the stationary phase through which they diffuse due to their molecular size differences. Large molecules elute quickly through the column as they are either totally or only partially excluded from entering the pores while small molecules penetrate deeper into the pores and therefore elute later [100]. In size-based separations using a set of known proteins and plotting the logarithm of the molecular weight vs. the retention volume allows the construction of a calibration curve that can be used for the estimation of the molecular weight of unknown proteins. In SEC the analysis time is determined by the flow rate of the mobile phase with a given column.

Increasing flow rate of the mobile phase or a reduction of the column length is a straightforward way to shorten analysis time; however, backpressure needs to be at a reasonable level when using high flow rates since it can affect stability of the packing material and resolution [98].

There are two main types of SEC columns: silica, with or without surface modifications, and cross-linked polymeric packings [101]. Comparison of three SEC columns with different particle sizes of 1.7 µm (ACQUITY UPLC BEH200 SEC), 3 µm (Zenix SEC-250) and 5 µm (TSKgel 3000 SWxl) for the analysis of antibody aggregates showed that the analysis time can be shortened using smaller particle sizes [102]. A column packed with sub-2 µm particles showed more than twofold improvement in throughput compared to the TSKgel column and further throughput was increased using parallel-interlaced mode allowing sample analysis in less than 2 min. Another report on the same with ethylene-bridged hybrid inorganic–organic (BEH) silica packing material with 1.7 µm particle size showed that high pressure and elevated temperature generated by small particles might cause on-column generation of additional protein aggregates [98].

The fractionation of different protein based on size reduces sample complexity that is particularly beneficial for top-down MS methods. However, some drawbacks of SEC including low resolution highlight the necessity for alternative methods. Ultra-high pressure fast size exclusion chromatography (UHP-SEC) has shown potential for rapid and high resolution separation of intact proteins [103]. UHP-SEC demonstrated fast separation of 6 standard proteins (BSA, ovalbumin, cytochrome C, aprotinin, thyroglobulin and IgG) in the mass range 6–669 kDa. Proteins were separated at flow rate of 0.2 mL min^−1^ in less than 7 min with comparable resolution and retention time using 50 mM sodium dihydrogenphosphate and a MS compatible solvent (50 mM ammonium acetate) [103]. Fractions collected with ammonium acetate were directly analyzed by ion cyclotron resonance Fourier transform MS without prior desalting and high resolution spectra confirmed protein molecular weights. Subsequent MS analysis of SEC fractions collected offline from MS is a useful tool for protein characterization [104]; however, coupling SEC online with MS minimizes the possibility of composition changes within the collected fractions prior to MS. One example is study of Munnerrudin et al. [105] where SEC was coupled online with native electrospray MS to characterize serum albumin, human transferrin and recombinant glycoprotein human arylsulfatase A. Proteins were dissolved in ammonium acetate and SEC separation was carried out using Biosuite ultra high resolution column. Online SEC-native ESI/MS enabled distinction between incompletely resolved proteins based on their mass differences and study of protein ion charge state distribution gave information on their conformational integrity.

The application of certain chromatographic techniques depends on protein-specific properties and the research aims. The implementation of different selectivities for protein analysis offers advantages and limitations where compromises between sensitivity, resolution, protein retention, mobile phase composition and the possibility of coupling separation online with MS, has to be made.

While significant progress has been made in developing new separation methodologies including modifications of stationary phases, implementation of MS compatible buffers and protein engineering, the challenge of intact protein analysis is yet to be conquered.

## Different Stationary Phase Morphologies for Intact Protein Separations

While different selectivities of stationary phases are often the first thing many analysts consider when developing analytical methods, the morphology, that is the structure, of the stationary phase is not always considered. Stationary phases can be packed with fully porous particles, the traditional option, core–shell particles, or non-porous particles. Additionally, the stationary phase may consist one a single porous rod structure (monolithic columns) or even coated on the walls of a capillary (open tubular). We will discuss these morphologies in relation to the analysis of intact proteins.

### Particle-Packed Columns

Stationary phase selectivity is an important factor governing resolution in chromatography. That said; the morphology of the stationary phase material is equally important. Unlike small molecules, where the eddy diffusion is arguably the predominant factor limiting the separation power, for proteins the rate of mass transfer dictates the separation power [106, 107]. A key factor governing the rate of mass transfer of proteins is the size of the pores of the stationary phase [106, 108–111]. When the pores of the stationary phase are too small relative to the hydrodynamic radius of the protein, they cannot enter the complete pore volume therefore experiencing a size exclusion chromatographic effect. The fraction of the total pore volume that is not accessible to pores is equal to (1 − (*R*_*H*_/*r*))^3^ where *R*_*H*_ is the hydrodynamic radius of the protein and *r* designates the radius of the pores [112]. Size exclusion of proteins in RPLC reduces the mass of protein that can be loaded onto the column but also the separation power, in terms of plate height (*h*). This has been examined experimentally and theoretically by a number of studies for what can be regarded as common model proteins: BSA, β-lactoglobulin, carbonic anhydrase isozyme, cytochrome C, IgG, insulin, lysozyme, myoglobin and ovalbumin. The results are in agreement; pores should be larger than the hydrodynamic radius of the proteins being separated [106, 108–111], at least 3 times as large [111]. Increasing the pore size from 90 to 160 Å increased the rate of mass transfer by up to 3.5 times for insulin, a relatively small protein of 5.6 kDa [106]. The benefit of large pore size was also seen for a large protein, BSA (66.8 kDa), as shown for the Aeris WIDEPORE column with 300 Å pore size. This stationary phase gave much better peak shape compared to stationary phases containing 160 and 175 Å, where peaks showed notable tailing [110]. The degree by which pore size limits the rate of mass transfer and in turn, *h*, increases with increasing size of the protein reducing the protein’s ability to access the internal pore volume thereby reducing external film mass transfer. While it is perhaps more intuitive to expect that the trans-particle mass transfer plays the primary role, the external film mass transfer was estimated to govern over 90 % of the overall mass transfer term of the van Deemter equation [106]. This means that larger pores increase the access of the protein and the mobile phase to the external surface area thereby increasing the rate of transfer of the protein through the film of stagnant mobile phase that coats the particles. This can lead one to believe that there is no benefit in using core–shell (otherwise known as superficially porous, partially porous or pellicular particles) relative to fully porous particles. However, numerous experiments have demonstrated the benefits of inclusion of a non-porous core [107, 111, 113]. In short, core–shell particles enable more efficient protein separations than fully porous particles. While the same trend noted above for pore size applies to both fully porous and core–shell particles [107, 111], the core–shell particles have the advantage of shortening the length through which proteins must diffuse during their migration through the column. This reduced distance arises directly from the inclusion of the non-porous core that is inaccessible to molecules. While this is not the primary advantage of core–shells in small molecule applications, where the reduction of eddy diffusion term plays the key role, it plays the dominant role in protein separations where the slow rate of diffusion of proteins compared to small molecules reduces their ability to undergo fast mass transfer [107, 111]. Studies that focused on developing core–shell particles specifically for protein separations found that the thinner the porous layer of the core-shell, the more efficient the separation provided that pore size and overall porosity is sufficient [111, 113]. Of course the thickness of the shell must be balanced with the need for a certain degree of mass loading capacity.

**Fig. 2:**
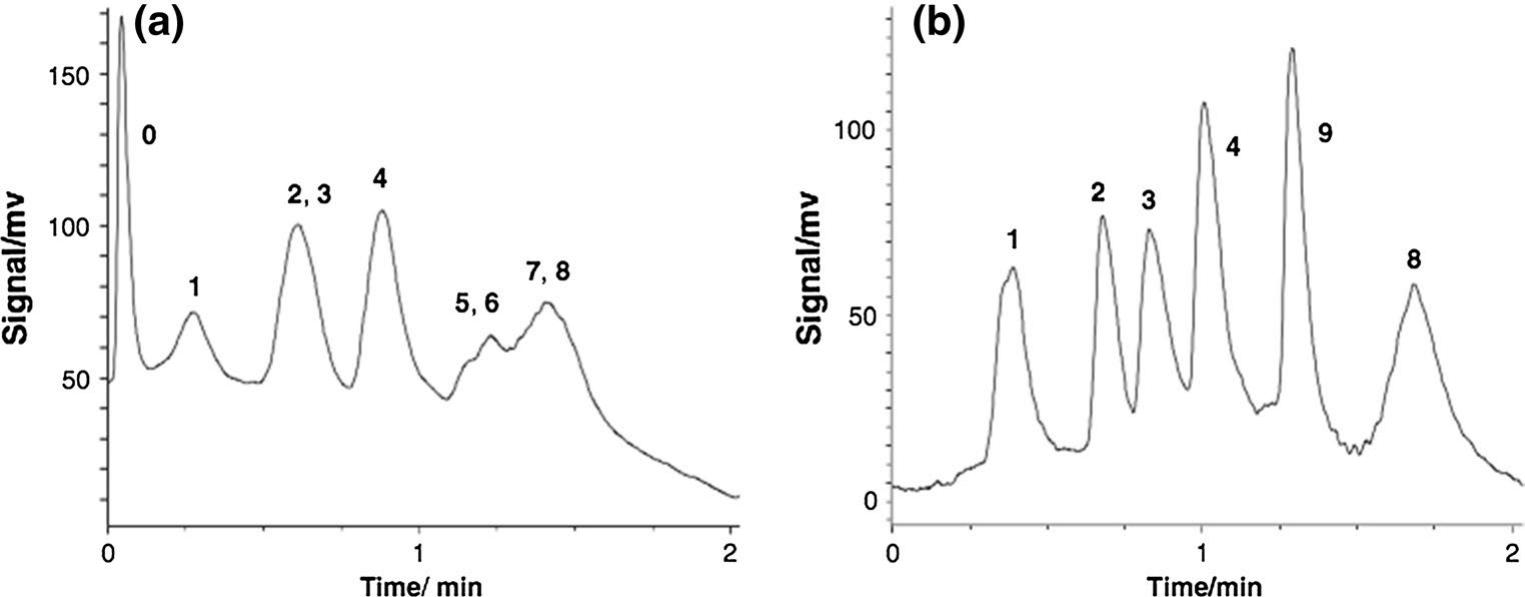
Separation of a mixture of intact protein standards: *1* Bovine heart cytochrome c, *2* Bovine pancreas ribonuclease A, *3* Equine heart myoglobin, *4* Chicken egg white lysozyme, *5*,*6* Bovine pancreas α-chymotrypsin, *7 Bacillus subtilis* α-amylase, *8* Bovine pancreas insulin using **a** a 10 mm i.d. × 2 mm chromatographic cake packed with 1.2 μm non-porous particles and **b** a 10 mm i.d. × 2 mm chromatographic cake packed with 630 nm non-porous particles. For both (**a**) and (**b**) the flow rate was 5 mL min^−1^ Reproduced with permission from [119]

When mass loading is not a concern because dilute solutions can be used with sufficient sensitivity, as is often the case in LC–MS, non-porous particles can be used. Although not widely used in practice, silica non-porous particles derivatized with C18 ligands have been applied for RPLC of intact proteins in mixtures of protein standards, antibodies, liver mitochondrial proteins, mouse liver extract, bovine endothelia cell membranes and human hepatocytes extract [114–119]. Because these particles do not contain pores, the resistance to mass transfer is eliminated resulting in reduced band broadening and consequently greater separation power, particularly for intact proteins which suffer significant broadening due to slow mass transfer as discussed above. This was demonstrated for the separation of liver mitochrondrial proteins where a 2 μm diameter non-porous silica-based C18 column was compared to a 3 μm, 300 Å wide-pore column. Both columns had the same dimensions and the same RPLC gradient elution programme and column temperature was used for each column. The non-porous column outperformed the wide-pore column resolving 420 protein peaks compared to 160 peaks for the wide-pore column [115]. Studies using non-porous columns have predominately used column lengths longer than 100 mm. Such columns produce significant backpressure due to the lack of permeability of the stationary phase. This necessitates the use of low flow rates resulting in long analysis times (as long as 999.8 min in one case [118]) despite the reduced retention due to their low surface area relative to porous particle columns. To get around this limitation a short (2 mm) yet wide (10 mm) “chromatographic cake” was employed [119]. The unusual dimensions of the cake allowed the use of 630 nm diameter non-porous particles without being encumbered by excessive backpressure. By using such small particles, the surface area of the cake was increased relative to a cake of the same dimensions packed with 1.2 μm particles. The benefit of using the 630 nm particles compared to the 1.2 μm particles was evident for the separation of a mixture of intact protein standards (Fig. 2) where 8 proteins were almost baseline resolved within 2 min. It should be noted that the flow rate used to produce such a fast separation was 5 mL min^−1^; such flow rates are not currently compatible with LC–MS. While the performance of chromatographic cake in terms of N may be limited due to its very short length, such columns may prove useful for producing relatively efficient separations when used as the second dimension of a two-dimensional comprehensive LC system where proteins enter the second dimension significantly diluted due to the high flow rates imposed on this dimension to produce extremely fast separations.

### Monolithic Columns

The presence of inter-particle void volume between the packed particles and the time required for diffusional mass transfer of solutes into and out of the mobile phase present in the pores of porous stationary phases are the major factors limiting the separation efficiency of porous packing materials, especially for proteins and peptides having low diffusivities [120]. Monolithic stationary phases were introduced in a search for new stationary phases with enhanced mass-transfer properties in which the separation medium consists of a continuous rod of a rigid, porous polymer that has internal porosity and no interstitial volume consisting of micro and macropores [121–124]. A key feature of these stationary phases is the presence of large through-pores, which enables the mass transfer to be driven mainly by convection, rather than by diffusion in pores of traditional particulate packing [125]. This accelerated mass transport is very valuable in the gradient separation of large molecules for which diffusion is slow. Hence, among the advantages of monoliths over packed materials in terms of chromatographic performance is the ability to achieve higher porosity that enables higher linear flow velocities and hence faster separations without a notable decrease in efficiency of separation. In comparison to particle packed columns, the monolithic ones display higher efficiencies at high flow rates [126]. Other advantages include the simplicity of their in situ preparation to create miniaturized capillary column formats.

Polymer-based monolithic chromatographic supports are usually prepared through the in situ polymerization of a mixture of suitable monomers and porogens within a tube that acts as a mold. Various precursors have been reported for the preparation of polymer-based monolithic stationary phases [126–129]. Monomers such as acrylamide [122], styrene and divinylbenzene [130, 131], acrylates [132], methacrylates [133–136] and norbornene [137] have been reported. The porous monolith can be covalently attached to the capillary wall increasing, by these means, the robustness of the column [138]. Bonding the monolith to the wall by surface-modification procedures was proven to be a crucial step, especially for large i.d. columns [139, 140]. The porous properties of the monolithic materials can be influenced by the composition of the polymerisation mixture by altering the ratio of porogens [141, 142] and the reaction conditions (polymerisation temperature and time) [143, 144]. Size and morphology of the pores strongly depend on several factors, including polymerisation kinetics and solvency of the porogens for the resulting polymer [145]. Tuning the morphology of the polymer-monolith is an important aspect to maximise the peak capacity.

**Fig. 3:**
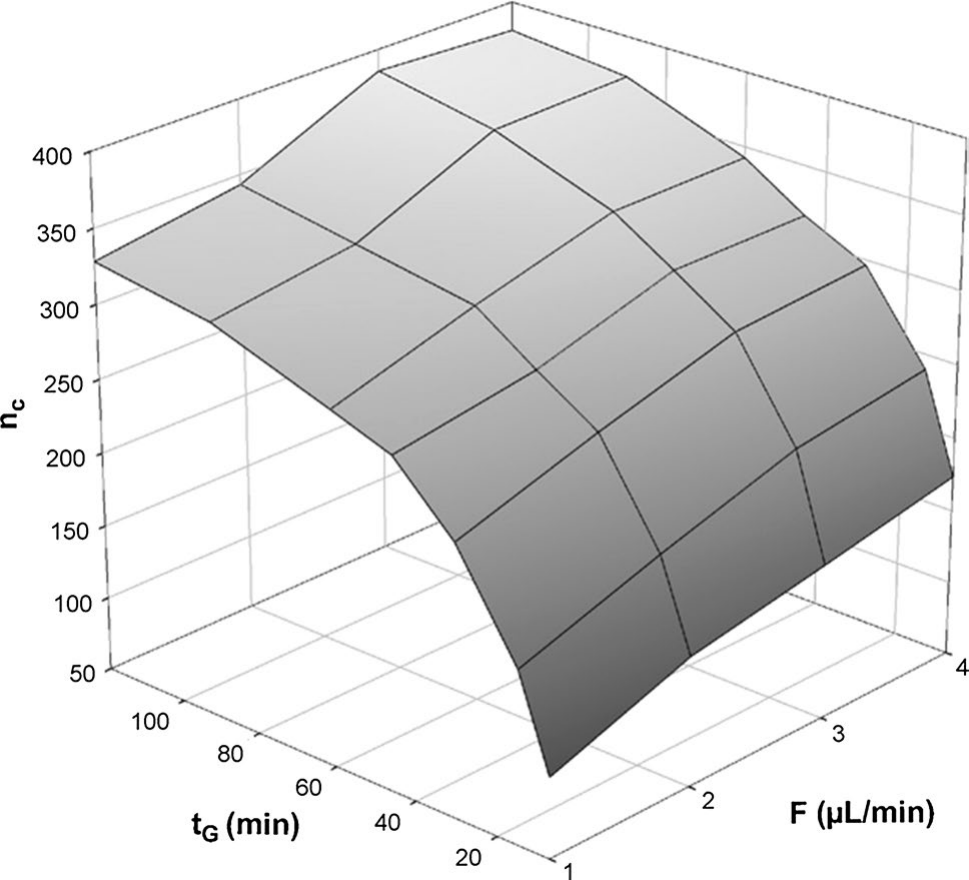
Effect of flow rate and gradient time on the peak capacity recorded on a 50 mm long monolithic column. Experimental conditions: mobile phase A = 0.05 % aqueous TFA, mobile phase B = 80:20 % ACN:H_2_O containing 0.04 % TFA. Gradient window from 15 to 80 % B. Injection volume = 1μL (0.6μg μL^−1^ per protein), Column temperature = 60 °C. Detection at 214 nm Reproduced with permission from [146]

The good separation performance of monolithic capillary columns with gradient elution has been demonstrated for complex mixtures of proteins [135, 146–148]. Detobel et al. [146], studied the effect of column parameters (morphology and length) and gradient conditions on the performance of capillary poly(styrene-co-divinylbenzene) monoliths. In agreement with the theory, the peak capacity increased according to the square root of the column length. It was also shown that decreasing the macropore size of the polymer monolith while maintaining the column length constant resulted in an increase in peak capacity. By using long (250 mm) monolithic columns with optimized morphology a peak capacity of 620 could be achieved for the separation of intact *E. coli* proteins using a 120 min gradient and UV detection. The maximum peak capacity obtained with shorter columns, 50 and 100 mm, were 330 and 440, respectively. The combined effects of flow rate and gradient time over the peak capacity are shown in Fig. 3. As shown, longer gradient times increased the peak capacities. At constant gradient times the peak capacities increased when increasing the flow rate. Eeltink et al. [149] reported the use of a 50 mm long poly(styrene-co-divinylbenzene) monolithic column (1 mm i.d.), operated at 90 μL min^−1^, and 80 °C, for the separation of an *E. coli* intact protein mixture by HPLC–UV. For a gradient time of 2 h a maximum peak capacity of 475 was obtained. In a more recent study, Eeltink et al. [148] studied the potential of long poly(styrene-co-divinylbenzene) monolithic capillary columns (250 mm × 0.2 mm) for the gradient elution of ABRF 48 intact protein standard mixture, including protein isoforms by LC–TOF–MS was studied. The separation of the 48 protein mixture using a gradient time of 120 min, at a flow rate of 1.5 μL min^−1^ and column oven temperature of 60 °C gave peak capacities >600. This allowed protein isoforms that differ only in their oxidation and biotinylation state, to be separated. However, protein identification, based on comparison of the experimentally determined molecular weight with theoretical masses was tentative, making it unsuitable for the analysis of actual biological samples. In total, 30 different protein masses were obtained from the 120 min gradient run. Based on molecular weight alone, only 24 charge envelopes could be tentatively assigned to proteins that are known to be in the 48 protein mixture. In another study, the use of a 50 mm long capillary poly(styrene-co-divinylbenzene) macroporous monolith (1 mm i.d.) using microLC-Orbitrap-MS setup showed limit of detection in the low femtomol range for a standard mixture of 9 proteins with a molecular weight ranging between 5.7 and 150 kDa. [150].

Recently, the use of strong cation exchange (SCX) sulfoalkylated monolithic cryogel for the separation of 3 model proteins was reported [135]. The continuous network comprising interconnected macropores (10–100 μm) gave little or no mass-transfer resistance allowing the use of high flow rates without losing separation power. In addition the ease of preparation make monolithic cryogels suitable media for separation of high molecular species [151]. The selected proteins were successfully separated using a linear gradient and the obtained chromatogram was compared to the one obtained with a non-functionalized cryogel column where the analytes coeluted. No evidence of irreversible protein adsorption was observed and the performance of the new synthesised material in chromatographic separations was found to be reproducible even after passing liters of eluents and 10–20 column volumes of 1 M sodium hydroxide. These results indicate that the novel cryogel-based monolithic columns should be further investigated for the separation and purification of proteins.

**Fig. 4:**
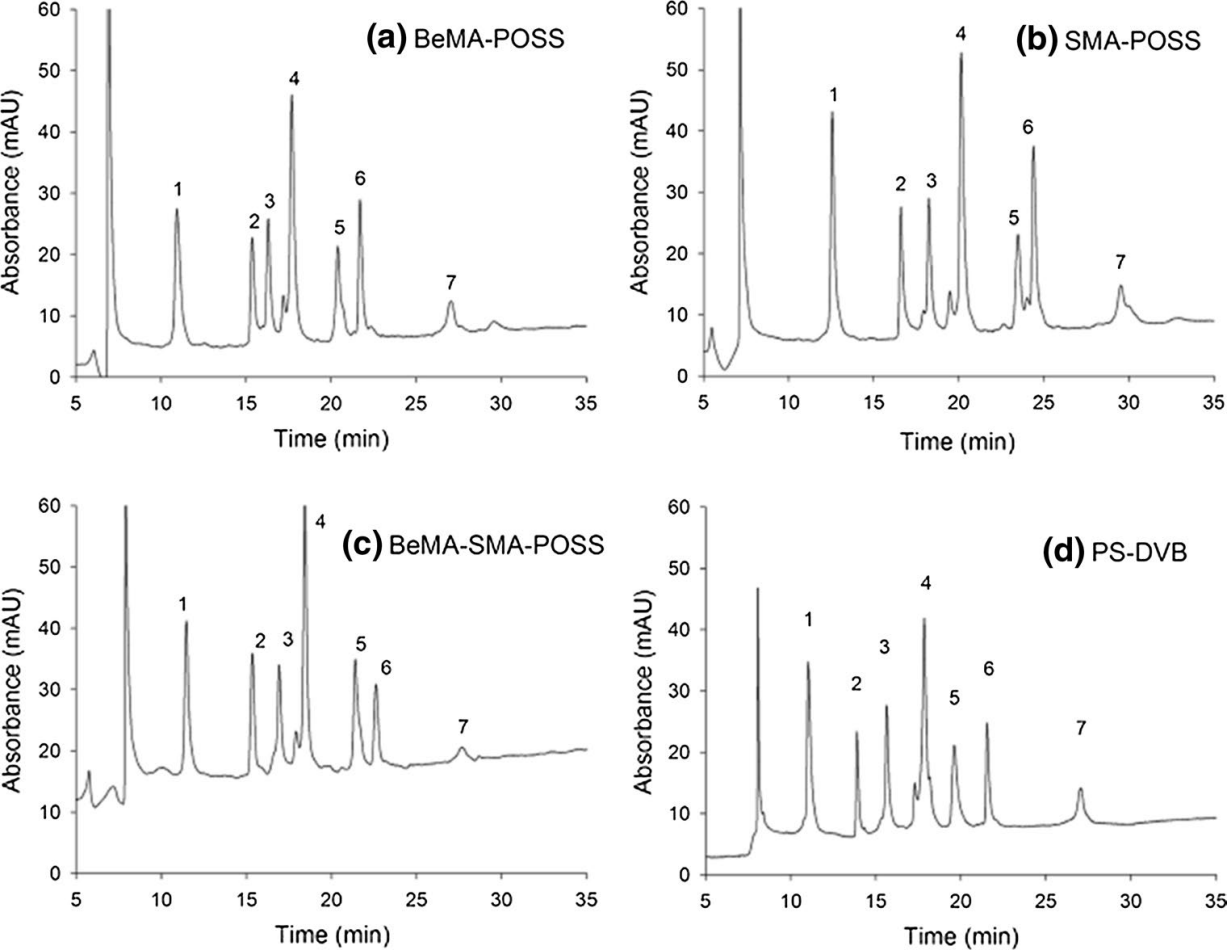
Chromatograms for the separation of intact protein mixture on the different monolithic capillary columns. **a** BeMA-POSS hybrid monolithic column, **b** SMA-POSS hybrid monolithic column, **c** BeMA-SMA-POSS hybrid monolithic column, **d** commercial PS-DVB monolithic column. Standard protein mixture: *1* ribonuclease B, *2* cytochrome c, *3* insulin, *4* lysozyme, *5* BSA, *6* myoglobin, *7* ovalumin. Injection, 1 μL of the standard protein mixture; flow rate, 500 nL min^−1^; gradient, 20–60 % B with 0.05 % TFA in 30 min; column, 100 μm i.d. × 25 cm; detection wavelength, 214 nm Reproduced with permission from [147]

A different approach for the separation of intact proteins was proposed by Liu et al. [147], using hybrid monolithic capillary columns based on polyhedral oligomeric silsequioxane and nano-LC with UV detection. The cage-like silsequioxane-polyhedral oligomeric silsesquioxane (POSS) were used as cross-linker for “one-pot” preparation of hybrid monolith columns embodying an inorganic–organic hybrid architecture with an inner inorganic framework. The authors compared the performance of the POSS-based hybrid monolithic columns for the separation of 7 standard proteins mixture and of *E. coli* proteins using gradient elution at 500 and 750 nL min^−1^, respectively. The results were compared with the ones obtained by analysing the same intact protein standard mixture by using a commercially available PS-DVB monolithic capillary column. The obtained chromatographs for the standard protein mixture using the three different monolithic stationary phases are shown in Fig. 4. As shown, the selected proteins showed stronger retention on the stearyl methacrylate-POSS (SMA-POSS) capillary columns than on the benzylmethacrylate-POSS (BeMA-POSS) ones, due to its higher hydrophobicity (Fig. 4a, b). Also a slightly different separation selectivity was observed. Based on these results the authors concluded that besides the hydrophobicity of the stationary phase and the π–π stacking interactions, introduced by the BeMA function monomer, may exert positive effect on the separation of some types of intact proteins. For this reason, a POSS based hybrid monolithic column was developed with an equal functional monomers SMA and BeMA was synthesized and tested for the same standard protein mixture (Fig. 4c). By using the SMA-BeMA hybrid monolithic capillary column, all the intact proteins were baseline separated. Peak capacities between 62 and 79 were reported for the standard protein mixture analyzed. The results showed that a combination of two functional monomers (stearyl- and -methacrylate) (BeMA-SMA-POSS) functional monomers presented a better LC separation selectivity than using one type only. Compared to the performance of the commercial PS-DVB capillary column, lower peak capacity was obtained with the SMA-BeMA hybrid monolithic column (79 and 68, respectively), but comparable run-to-run reproducibility (approx. 1 %).

### Open-Tubular Columns

Open tubular (or open channel) chromatography (OTLC), initially developed by Halasz and Horvath [152], offers a significant gain in column efficiency compared to packed columns in terms of time and separation, which has been demonstrated theoretically and empirically [153]. Their performance is directly connected to the internal diameter; efficiency increasing with decreasing inner diameter [154]. In order to achieve efficiencies comparable to those of good packed columns, porous layer open tubular (PLOT) columns must have an inner diameter of the order of 15 μm or less. Jorgenson and Guthrie [155] published the first report on open tubular columns with an inner diameter close to what is theoretically required for efficiency similar to packed columns (~15 μm). Recently, 10 μm i.d. PLOT polystyrene divinylbenzene (PS-DVB) columns have been designed and used for high resolution, ultratrace LC–MS separations of peptides [156, 157]. Causon et al. [158] performed a kinetic optimisation of OTLC capillaries coated with thick porous layers taking into account the effect on the retention, column resistance, band broadening and mass loadability. Their calculations showed the need to develop coating procedures that will produce porous films filling up approximately 50–70 % of the total column diameter offering very good reduced plate heights (*h*_min_ < 2 for *k*’ = 3). In the same study it was shown that by using elevated temperatures (90 °C) the allowable column diameter can be increased up to 9 μm (for lengths >0.8 m) achieving a large range of N values (100,000–880,000), and hence presenting an advantage over packed LC columns.

**Fig. 5:**
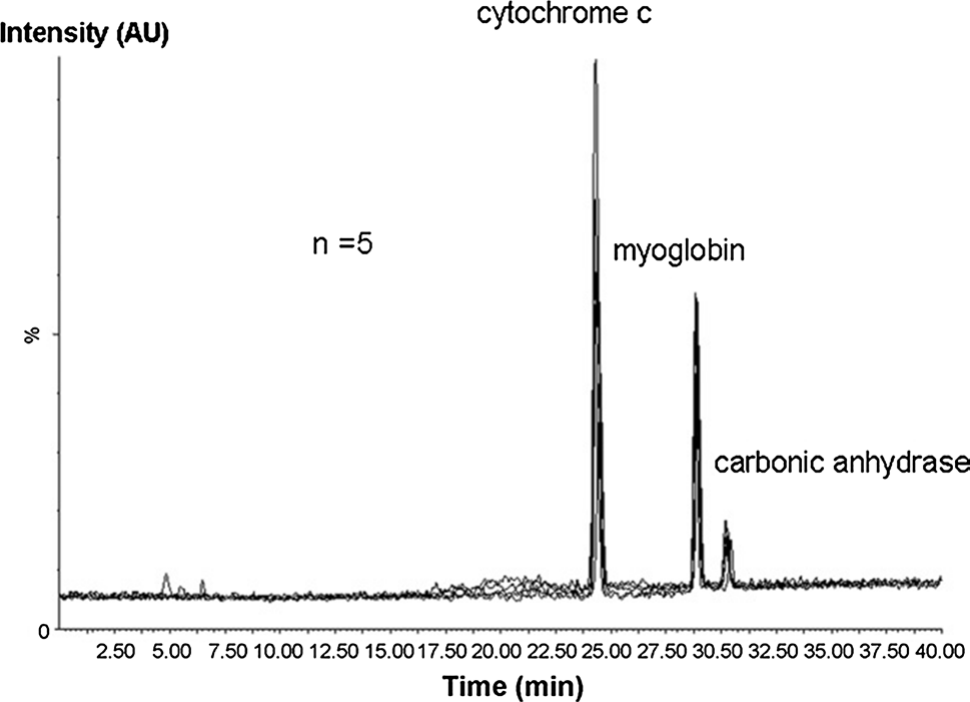
Overlay of five consecutive separations of three standard proteins (33 μg mL^−1^ each) with PLOT nanospray-MS (10 μm i.d. × 3 m) under gradient elution (90 % A (0.1 % FA, 0.05 % TFA (v/v) in water) to 90 % B (0.1 % FA, 0.05 % TFA, 10 % water (v/v) in ACN) in 40 min) Reproduced with permission from [160]

**Fig. 6:**
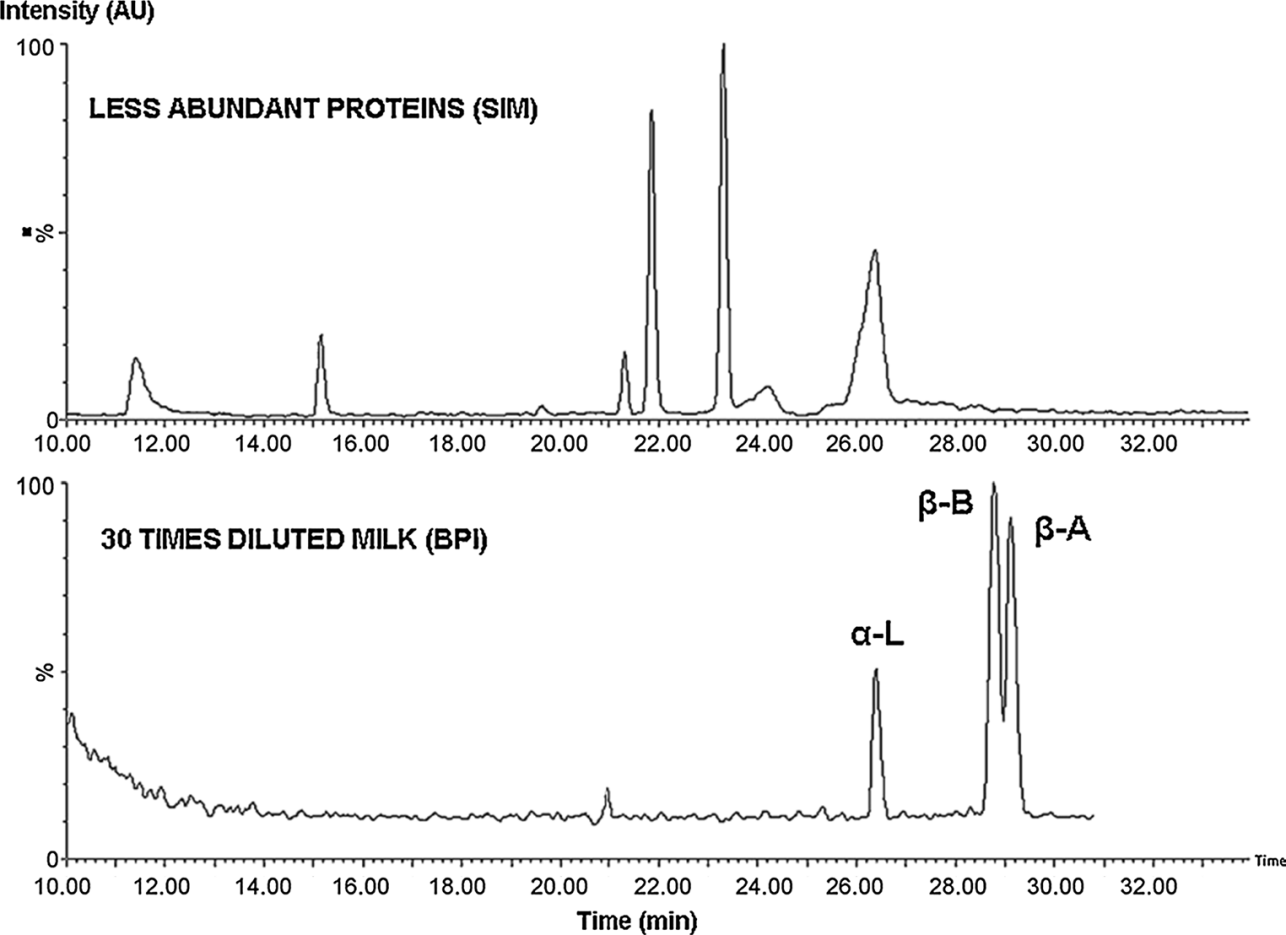
Separation of proteins in skimmed milk. *Top figure* is a combined selected ion monitoring (SIM) of less abundant proteins (1.5 times diluted milk), *bottom figure* is a BPI, showing separation of main proteins α-lactalbumin, β-lactoglobulin B and β-lactoglobulin A (30 times diluted milk). Conditions were as in Fig. 5 Reproduced with permission from [160]

The use of PLOT columns for LC gained renewed interest after the coupling to nanospray-MS proving to be simple and efficient [159]. Their extremely small volumes require small injector and detector volumes. Such columns can be operated at low nanolitre flow rates and easily interfaced with ESI–MS, producing smaller droplets and thus minimising ion-suppression and yielding improvements in sensitivity [156]. PLOT columns were used for the analysis of intact proteins in a few studies [160, 161]. Kazarian et al. [161] reported the use of wall-modified with photonic crystal fiber based PLOT capillary columns with polystyrene-divinylbenzene (PS-DVB) porous layer for the analysis of cytochrome c under isocratic conditions obtaining run-to-run retention time reproducibility of below 1 %. The columns consisted of 126 internal parallel 4 mm channels, each containing a wall bonded porous monolithic type PS-DVB layer in PLOT column format. Rogerberg et al. [160] investigated the potential of PS-DVB PLOT columns (10 μm i.d. × 3 m) for the separation of three intact proteins (cytochrome C, myoglobin and carbonic anhydrase) by LC-nanospray MS under gradient elution (Fig. 5). They provided narrow peaks (approx. 0.2 min), very low carry-over (<1 %) and good repeatabilities (relative standard deviation (RSD) below 0.6 % and below 2.5 %, respectively). The effect of column length (1–3 m), gradient time (20–120 min) and column temperature (20–50 °C) was investigated. With shorter columns (1 and 2 m), peak widths were larger and increased more steeply with gradient time. Theoretical peak capacity (*n*_c_) increased with column length. The *n*_c_ increased with *t*_G_ until a plateau was reached. The highest peak capacity achieved (*n*_c_ = 185) was obtained with a 3 m column, where a plateau was reached with gradient time (*t*_G_) 90 min. A decrease in retention and increase in selectivity was observed with the increase in temperature. Studies of retention in relation to temperature indicated that the stationary phase undergoes changes at high temperatures. Peak heights decreased as a function of temperature which was attributed to a poorer charged droplet formation at elevated temperatures. The developed method was successfully applied for the analysis of intact protein in skimmed milk (0.1 % fat). Figure 6 presents the selected ion monitoring chromatogram (SIM) of lower abundant proteins in milk and the chromatogram obtained for 30 times diluted milk sample of highly abundant proteins.

## Emerging Chromatographic Technologies

To successfully address complex separation problems in proteomics, the development of novel separation technologies capable of achieving ultra-high peak capacities within a reasonable time allowing the analysis of a multitude of samples is crucial. There are a number of new chromatographic technologies that have shown promise for small molecules, peptides and in some cases, intact proteins, but have yet to be widely applied within the field of proteomics. In the interests of directing the reader towards the latest technologies for intact protein separations, we will briefly review three techniques that have demonstrated promise but have yet been widely accepted by the proteomics community.

### Slip Flow Chromatography

**Fig. 7:**
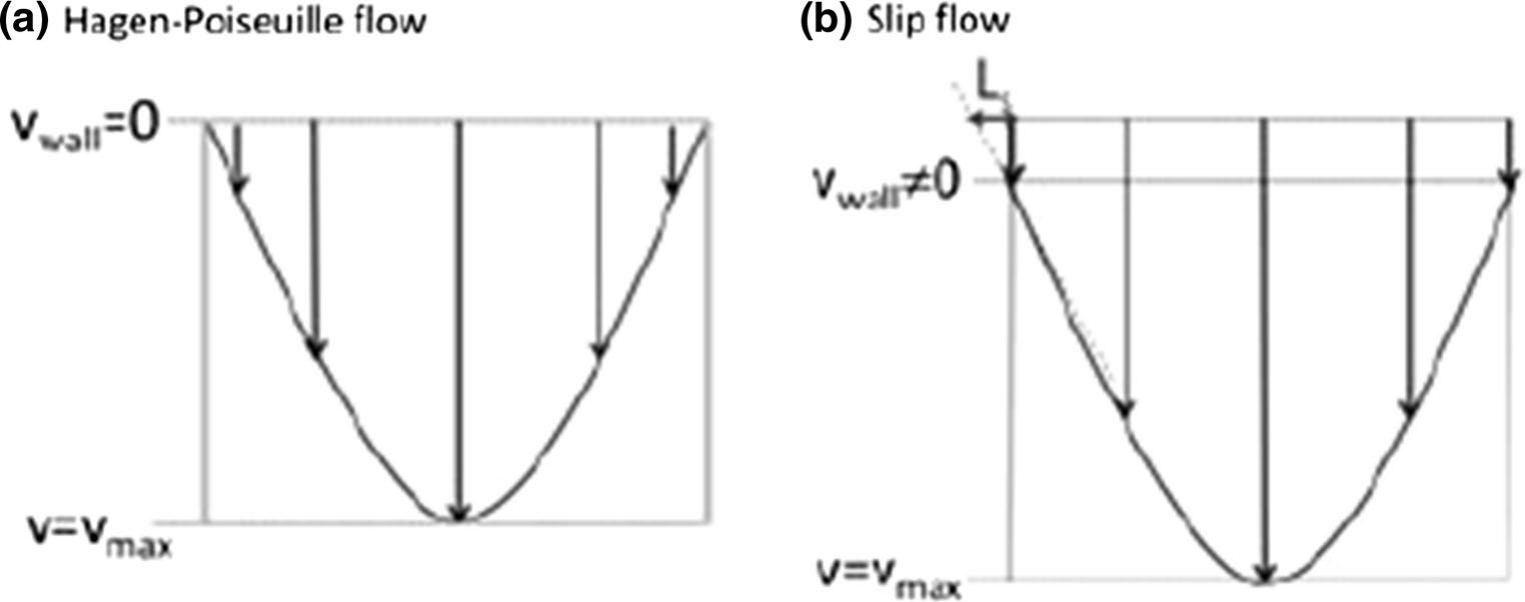
**a** Illustration of a Hagen-Poiseuille flow profile compared to (**b**) and illustration of a slip flow profile. L corresponds to the slip length which is an additive factor which imparts a non-zero velocity at the wall Reproduced with permission from [167]

Slip flow is a new variant of liquid chromatography. Essentially the different between slip flow chromatography and conventional liquid chromatography lies in the way solute bands migrate through the column. In conventional chromatography it has been well-established that solute bands migrate with a Hagen-Poiseuille flow profile. That is, solute bands can be thought of as migrating through the column with a profile somewhat resembling an empty bowl. This arises from many, well-documented factors [162, 163]. One of which, is that due to friction the velocity of the mobile phase at the wall approaches zero. However, in theory it is generally accepted to consider the flow velocity as equal zero due to strong interactions at the wall with solvent molecules, despite the fact that this is not exactly the case in practice. Conversely, in slip flow chromatography the velocity of the mobile phase is not zero. This is because the flow does not follow Hagen-Poiseuille principles but slips by the column wall due to weak interactions between the mobile phase molecules and the wall itself [164–169]. Figure 7 illustrates the difference between slip flow and Hagen-Poiseuille flow. The benefit slip flow is that the solute band is less distorted than in Poisieuille flow conditions due to a more homogenous radial flow velocity profile. Consequently, the peaks eluting from the column in slip flow are more Gaussian compared to the Poisieuille flow case. Furthermore, the flow enhancement arising from slip flow allows sub-micrometer particles to be operated in columns without generating unreasonable pressure [164–169]. The use of smaller particles further increases chromatographic efficiency, as we know from the van Deemter equation. It is important to note that certain experimental conditions are required to generate slip flow in packed columns. Firstly, the fluid passing through the column must show weak interactions with the column wall. This has been demonstrated using a column packed with silica particles with C_4_ ligands and particle diameters ranging from 125 to 1300 nm [165, 167]. The flow rate of toluene passing through this column was compared to the flow rate when water was passing through the column using the Kozeny-Carman equation, which is known to described the flow velocity of fluids through packed bed (Eq. 1)

1 $$ \frac{P}{L} = \frac{180 \cdot \nu \cdot \eta }{{d_{\text{p}}^{2} }}\frac{{(1 - \varepsilon )^{2} }}{{\varepsilon^{3} }} $$

where *P* denotes the pressure, *L* is the column length, *ν* is the flow velocity, *η* is the viscosity of the fluid passing through the packed bed, *d*_p_ is the particle diameter and ε is the porosity. Accounting for the viscosity differences between toluene and water, the latter produced a flow rate that was higher than the expected velocity according to Eq. 1 because water molecules had weaker interactions with the hydrocarbon column wall compared to toluene [165, 167]. Another parameter important for slip flow is the particle diameter which is related to the hydraulic radius, *r*_hyd_, by Eq. 2.

2 $$ r_{\text{hyd}} = \frac{{d_{\text{p}} }}{3}\frac{\varepsilon }{(1 - \varepsilon )} $$

It was found that as the particle diameter decreased from 1300 to 125 nm, the flow enhancement due to slip flow increase exponentially with a flow enhancement as high as 20 for 125 nm particles [165, 167]. However, simulations of slip flow in packed beds with colloidal silica particles predicted a much reduced flow enhancement than the experimental results described above [166, 167]. While the theory underlying slip flow in open capillaries is understood, the theory behind slip flow in packed beds has recently been investigated. As such the discrepancy between the experimental and simulated results is not definitively understood as yet. That said, simulations of flow moving through the packed bed indicated that regions of stagnant flow exist where the fluid stream makes contact with the particles indicating that tortuosity may affect the flow velocity profile in slip flow chromatography thereby reducing the flow enhancement [166, 167].

Even though the theory underpinning slip flow chromatography is still being formulated it has proven itself effective for the separation of intact proteins. A preliminary investigation using 2.1 cm column packed with C4 functionalized colloidal silica crystals and fluorescence detection showed extremely high efficiency for bovine serum albumin; higher than 1,000,000 theoretical plates [164]. The ability of slip flow chromatography to separate intact proteins was investigated in a follow-up study combining slip flow chromatography with LC–MS [168]. A 4 cm long column with a pulled tip, packed with 470 nm silica particles with C18 ligands was used in conjunction with ESI–MS for the analysis of model proteins, namely ribonuclease A, trypsin inhibitor and carbonic anhydrase [168]. Despite the small particle diameter of the stationary phase, the separation was performed with at a flow rate of 200 nL min^−1^ which is sufficiently high for LC–MS. The backpressure was 600 bar which is surprising reasonable given the particle size. This is a result of the flow enhancement effect under slip flow conditions. The LC–MS separation showed high efficiency with peak capacity of 195 for a 10 min gradient [168]. This high resolving power facilitated the identification of four proteoforms for the ribonuclease A and carbonic anhydrase standards. Two proteoforms were identified for superoxide dismutase and trypsin inhibitor. The presence of proteoforms in commercial protein standards is known to increase the peak width of intact proteins separations as the proteoforms are very hard to separated and typically co-elute. The ability of slip flow chromatography to facilitate the identification of different proteoforms by MS illustrates its potential for proteomics despite that fact that to date it has only been demonstrated for model protein separations. That said, the colloidal silica used in slip flow columns is non-porous therefore they share the same limitation that is encountered when using columns packed with non-porous particles; the relatively low surface area reduces the retention of solutes and reduces the mass that can be loaded onto the column. Attention should also be paid to the interactions between the capillary wall and the mobile phase. As discussed above, interactions between the wall and the mobile phase must be weak in order to generate slip flow. While this may not be an issue for RPLC as the solvent is polar, it may become an issue for more non-polar solvents for example, in HILIC or normal phase LC. This may be alleviated by coating the wall so it becomes less hydrophobic like what has been reported in capillary electrophoresis [170–175].

### Microfluidics

Recently many common analytical assays, including DNA separation, cell manipulation and protein analysis have been reduced in size and manufactured in cm–scale devices as an alternative to the column-based approach [176]. These devices are called microfluidics and are analytical tools where fluids are driven (hydrodynamically or electrokinetically) through microstructured channels. They incorporate different functions required to analyse a particular sample (e.g., injection loop, stationary phase, valve, detector, etc.) into a single platform providing high levels of process automatization. This enables the construction of miniaturized analytical tools often called “lab on a chip” devices.

**Fig. 8:**
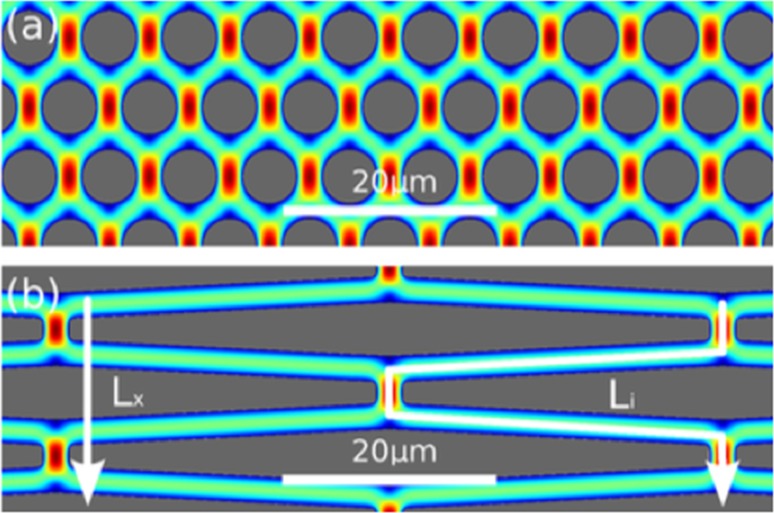
Illustration of **a** a section of a cylindrical pillar array column compared to **b** a section of a radially elongated pillar *array column*. *Colours* indicate the flow velocity as simulated using computation fluid dynamics software (namely, Comsol version 4.3). The flow direction is shown by the *white arrow* labelled *L*_x._ The *white arrow* labeled *L*_i_ shows the direction of flow via the tortuous path followed by the mobile phase Reproduced with permission from [179]

The main advantages of using microfluidic devices for separations than conventional LC in a column are the low dead-volume connections that minimize band-broadening effects, the ability to analyse ultra-low sample volumes, reduced solvent consumption and operating costs. Furthermore, short analysis times can be achieved due to the reduced length scales without sacrificing efficiency producing high peak capacities. In addition, they can integrate multiple sample preparation steps into one device. The types of stationary phase morphologies that can be used for liquid chromatography microfluidic platforms are (1) open-tubular channels, (2) microfabricated pillar-array columns, (3) packed particles and (4) polymer monoliths. Liu et al. [177] reported the application of a microfluidic device with an integrated solid phase extraction segment coupled to a 15 cm column packed with polymer monolith for the separation of labelled intact proteins within 15 min, which is fast considering the low flow rate was only 200 μL min^−1^. Taniguchi et al. [178] reported a polydimethylsiloxane microfluidic chip-based approach for the quantitation of *E. coli* proteome with single molecule sensitivity. This level of sensitivity is important in facilitating the study of gene expression and regulation of low abundance proteins. Recently, Desmet et al. [179] reported the development of a micro-fabricated packed bed column filled with radially elongated pillars (Fig. 8) showing the same performance as non-packed open tubular columns. As previously mentioned, these offer a much higher separation speed and efficiency than conventionally packed bed columns. Efficiency in terms of N was as high as 70,000 plates for retained coumarin dyes. However, it should be noted that these dyes are small molecules and as such their separation efficiency is primarily limited by eddy dispersion, which is virtually non-existent in radially elongated pillar columns due to the high degree of order within the separation bed. As discussed in “Particle-Packed Columns”, unlike small molecules, the ability to separate proteins with high efficiency is predominantly limited by their slow rate of diffusion which increases the time taken for mass transfer. The radially elongated pillar columns should also compensate for slow mass transfer as they are non-porous, therefore we expect that the efficiency observed for intact proteins separations using non-porous columns (“Particle-Packed Columns”) should be comparable to the potential power of pillar columns.

Although microfluidic devices showed several promising applications in the proteomics field, there are still various challenges that need to be addressed before they gain wide acceptance for intact protein analysis. First, assays that show higher sensitivity must be developed. The most interesting proteins are present at very low concentrations and cannot be measured by antibody-based approaches and the majority of the studies were carried out using highly concentrated protein mixtures or model biological samples. Second, it is imperative to translate the proof-of-principle experiments into robust and easy to use methods that biologists and analytical chemists in the biomedical field would adopt. Moreover, pre-treatment and post-analysis elements are expected to be incorporated resulting in automatic and user-friendly systems.

### Active Flow Technology

**Fig. 9:**
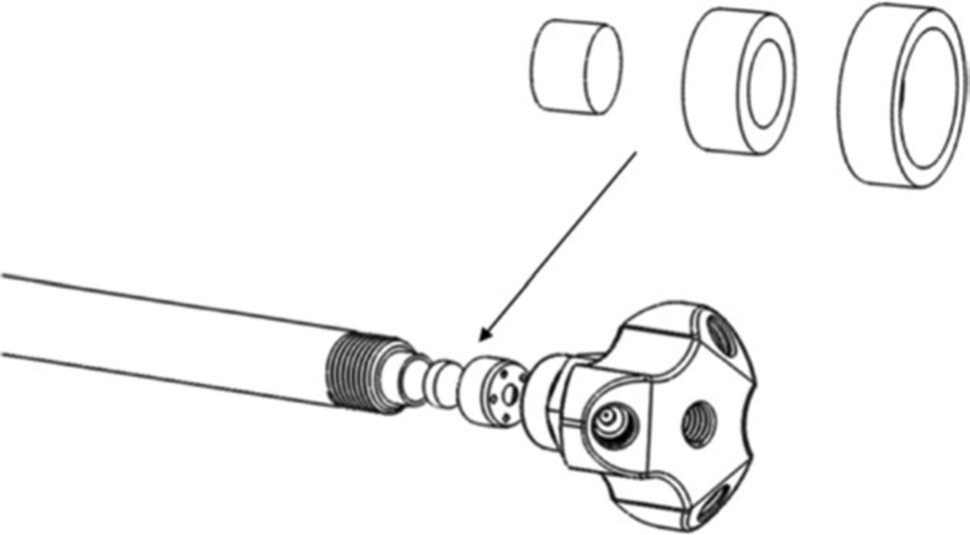
Illustration of the AFT column end fitting. The end fitting consists of an annular frit and a multi-port end cap. Flow is split through the annular frit, which is expanded to illustrate its design. Once the flow has been segmented via the frit it is diverted out of the various ports. The radial section of the solute band exits the central port whilst the wall-region portion of the band exits from the peripheral ports. The ratio of flow exiting the ports with respect to each other can be altered by altering the amount of backpressure using tubing typically connected to the peripheral ports Reproduced with permission from [183]

In an effort to increase sensitivity, capillaries packed with particles or containing monolithic beds are used to reduce the dilution of the sample. Capillaries typically range in size from 50 μm to as large as 1 mm i.d. Flow rates in the order of hundreds of nL min^−1^ are employed with these columns which makes them compatible for coupling with MS; low flow rates facilitate the removal of mobile phase during ionization using ESI. However, the need to use low flow rates increases the time required for analysis, which is undesirable, particularly when numerous degradable samples must be analyzed in a given day. Active flow technology (AFT) allows the use of wider i.d. columns at high flow rates (in the order of mL min^−1^) without compromising the MS. Separations of amino acids in fruit and vegetable juices have been accomplished within 24 s using AFT with LC–MS [180]. The ability of AFT columns to enable fast separations with MS lies in the unique design of end fitting used with these columns (Fig. 9).

The AFT end fitting consists of four ports. The central port is connected to the detector, or MS, while the flow from the remaining exits is typically directed to a waste reservoir. This end fitting was designed to minimize the effect of the heterogeneity of the stationary phase bed, which has been long known to cause band-broadening due to the difference between the mobile phase velocity in the radial centre of the bed compared to that of the mobile phase travelling near the wall of the column, which is slower. This radial velocity difference imparts a bowl-like flow profile to the solute band rather than a flat disc profile. The AFT fitting uses a novel frit design to convert the broader, bowl-like band to a flat disc profile using what could be equated to a cookie cutter: the inner frit, in essence, cuts the flat, disc-like part of the solute band which exits via the central port and travels to the MS. The flow from the tailing wall-region of the solute band exits via the remaining ports, commonly referred to as the peripheral ports, and is diverted to waste. This produces a more Gaussian, narrow peak which translates to higher efficiency, in terms of N [181–186]. Gains in N as high as 70 % relative to conventional columns have been reported for separations of small molecules [184]. However, a recent study comparing the results of AFT for separations of small molecules compared to larger molecules, namely insulin, has shown that the improved separation power reported for small molecules does not occur for large molecules [187]. As previously discussed, the efficiency of protein separations are primarily limited by slow mass transfer. AFT does not have any effect of the mass transfer properties of the stationary phase: AFT columns are packed with the same procedure and materials as conventional chromatography columns. The benefit of AFT for small molecules arises from the reduction of the long-range eddy dispersion [186, 187]. Where AFT is of use for proteins lies primarily in its ability to operate at fast flow rates thereby shortening the required analysis time. This is accomplished by controlling the flow rate from each port by applying different amounts of backpressure using tubing connected to the peripheral ports. The ratio of flow from the central port relative to the total flow rate is often referred to as the segmentation ratio, which is a useful means for tuning the performance of the AFT column depending on the specific application. For detailed information on how the segmentation ratio affects the performance of the column, readers are directed to the following references [184, 188]. To achieve fast separations within 24 s for amino acids in fruit and vegetable juice [180], the total flow rate was 4.5 mL min^−1^. Of this total flow rate, 21 % of the flow was sent through the central port to the MS. This equated to a flow rate of 0.9 mL min^−1^ to the MS. The column (50 mm × 2.1 mm i.d) was packed with 5 μm, fully-porous particles with C18 selectivity. The performance of the AFT column was compared to that of a conventional column (30 mm × 2.1 mm i.d.) with the same selectivity but packed with 1.9 μm fully-porous particles. This column more closely represents the current state-of-the-art for fast separations: short columns with small particles operated with UHPLC instrumentation to enable high flow rates despite the high backpressure. As expected, the column with 1.9 μm particles was more efficient at its optimal flow rate than the AFT column packed with 5 μm particles at its optimal flow rate. However, when the 1.9 μm particle packed column was operated at a flow rate (~0.9 mL min^−1^) near the maximum possible flow rate, the analysis time required for the same separation was ~70 % longer than that using the AFT column at a flow rate that gave the same value for N. Recently separations as fast as 12 s have been reported for monitoring the degradation of amino acids by nitric acid using AFT with LC–MS [189]. While flow splitting using a t-piece connection can be used as a means of operating fast separations yet reducing the flow rate sent to the MS, a comparison of this approach with AFT has shown that the AFT flow splitting is more efficient [190]. This is because the frit used in the AFT end fitting allows the central portion of the solute band to be separated from the wall region of the bowl-like solute band preventing dilution. This is not possible with a t-piece connection as such a connection includes the entire bowl-like solute band including the mobile phase within the hollow centre of the band prior to splitting the flow.

To date all studies using AFT have focused on small molecules rather than large molecules. While the benefits of operating at high flow rates to reduce analysis time should also be evident for proteins, it is possible that the slow mass transfer of proteins, which exerts a more dominant effect on N at higher flow rates, may reduce the benefit of operating at such flow rates. One way to compensate for this may be to combine the benefits of non-porous particles with AFT.

## Conclusion

The study of the human proteome is key to a better understanding of the various biological processes that take place within our bodies. Potentially this may lead to the identification of biomarkers for a variety of diseases. However, this requires powerful separation techniques to combat the complexity and variation in abundance of various proteins. LC–MS is arguably the most well-used tool in the field of proteomics, combining the separation powers of LC and MS to enable identification and characterization of proteins. In conjunction with top-down proteomics, where proteins are kept intact, the complexity and dynamic range of the proteome can be reduced further enabling more effective characterisation using MS.

To date RPLC using C18, C8 or C4 fully porous particle packed columns remain the workhorse in proteomics. Developments in column technology have not been readily adapted into the field. That said, the potential of core–shell particles, non-porous particles, monolithic columns and in particular slip flow chromatography have demonstrated potential for the analysis of intact proteins. Emerging technologies such as microfluidics and active flow technology may also prove to be valuable for reducing analysis and time and increasing sensitivity. Relatively new retention mechanisms such as HILIC show promise as they are able to retain polar proteins and the high organic modifier content of the mobile phase improves electrospray ionization. A closer link between chromatographers and biologists in the future may help introduce these developments into proteomics where they can be of great use in improving our understanding of disease and our biology.
